# Cystine/Glutamine Mixture Supplementation Attenuated Fatigue during Endurance Exercise in Healthy Young Men by Enhancing Fatty Acid Utilization

**DOI:** 10.3390/sports10100147

**Published:** 2022-09-27

**Authors:** Sihui Ma, Miho Ono, Ami Mizugaki, Hiroyuki Kato, Masashi Miyashita, Katsuhiko Suzuki

**Affiliations:** 1 Faculty of Sport Sciences, Waseda University, Tokorozawa 3591141, Saitama, Japan; 2 Japan Society for the Promotion of Science, Chiyoda-ku 1020083, Tokyo, Japan; 3 Institute of Food Sciences and Technologies, Ajinomoto Co., Inc., Kawasaki 2108680, Kanagawa, Japan

**Keywords:** amino acid supplementation, cystine/cysteine, glutamine, exercise, endurance

## Abstract

Exercise-induced fatigue is a multi-origin physical and mental phenomenon. Efforts to diminish the above predisposition may contribute to endurance, along with athletic well-being, while development of nutritional strategies to optimize condition and exercise performance are essential issues for athletes and trainers. Dietary amino acids are being discussed for their specific health-promoting properties beyond their role as building blocks of proteins. Glutamine, along with cysteine, are two kinds of amino acids that are reported extensively for their anti-oxidation, anti-inflammation, and immune-regulation properties, and are promising in sport applications. In the present study, we designed a randomized, placebo-controlled, crossover trial to examine effects of 7-day supplementation of cystine/glutamine mixture (Cys2/Gln) on self-reporting fatigue index (ratings of perceived exertion, RPE), energy metabolism, and inflammation. We also employed a C2C12 myotube model to examine the capacity of cystine for fatty acid utilization. Cys2/Gln supplementation alleviated fatigue by decreasing RPE and enhanced fatty acid oxidation during a 60 min endurance exercise in human trials, while cystine increased fatty acid utilization in C2C12 myotubes by enhancing mitochondrial respiration. In summary, Cys2/Gln supplementation exerts positive effects on ameliorating exercise-induced fatigue, mechanisms of which can be attributed to enhancement of fatty acid utilization.

## 1. Introduction

Fatigue is a symptom that can be associated with a state of exhaustion, following strenuous activity or physical exercise. At present, deletion of energy substances [1], accumulation of metabolites [2], production of reactive oxygen species (ROS) [3,4], and up-regulated inflammatory reaction [5] are all considered to be the factors that lead to fatigue. Fatty acid utilization capacity, including fatty acid mobilization and fatty acid oxidation (FAO), can also contribute to delay fatigue, and is deeply associated with exercise performance, especially endurance [6]. In addition to training, dietary intervention has been extensively applied to enhance exercise capacity based on these principles and hypotheses [7]. Specific nutrients, for instance, glutathione (GSH), are reported to enhance endurance by increasing FAO potential or quenching ROS [8]. Therefore, monitoring free fatty acid metabolism may be applicable for evaluating specific nutrients’ potential toward FAO capacity.

Dietary amino acids are being discussed for their specific health-promoting properties beyond their roles as building blocks of proteins, including relieving fatigue and improving exercise performance [9]. Of common amino acids, cysteine and glutamine are garnering attention for their potential to improve exercise performance by ameliorating exercise-induced inflammation and oxidation, preserving gastrointestinal (GI) function, and maintaining the stability of immune function followed by strenuous exercise [10,11,12,13,14,15,16], therefore, enhancing exercise performance and contributing to athletes’ conditioning.

Cysteine, along with its oxidized dimer cystine, possess the antioxidant ability of donating a hydrogen from their thiol groups [17], and play key roles in regulating cell redox state. During exercise, muscle-derived ROS contribute to fatigue, while fatigue could be delayed by administration of ROS-specific antioxidant N-acetylcysteine [18]. Glutamine is a non-essential amino acid of the glucogenic amino acid family. As a major fuel for immune cells, glutamine is reported extensively for its regulating roles on T-lymphocyte proliferation [13], cytokine and antibody production [14], macrophage activation [15], and essential cell energy metabolism as a component of the malate shuttle and tricarboxylic acid (TCA) cycle [16]. Cysteine and glutamine are both precursors of GSH [19]. In antigen-processing cells, for instance, T-helper cells, adequate GSH is essential for sustaining immune cell function [11]. Deficiency of extracellular cysteine or intracellular GSH are reported to impair the proliferation of lymphocytes and secretion of cytokines, thereby diminishing immune defense [12,13]. In addition, GSH supplementation has been reported to improve lipid metabolism by enhancing FAO in ICR mice [9].

It is established in both animal models and human trials that both cysteine/cystine and glutamine exert effects on immune modulation and GI function preservation, mechanisms which are mainly attributed to the anti-inflammatory and anti-oxidative properties, indicating that these amino acid supplementations may contribute to exercise capacity by ameliorating fatigue, through anti-inflammation and/or anti-oxidation effects [20,21,22,23,24]. Therefore, in the present study, we designed a randomized, placebo-controlled, crossover trial to examine a 7-day supplementation of cystine/glutamine mixture (Cys2/Gln) on exercise metabolism focusing on FAO and perceived exertion during exercise. Furthermore, to verify our results, we also employed a C2C12 myotube model to examine the capacity of cystine for mitochondria respiration and FAO.

## 2. Materials and Methods

### 2.1. Experimental Design and Experimental Protocol

**Figure 1:**
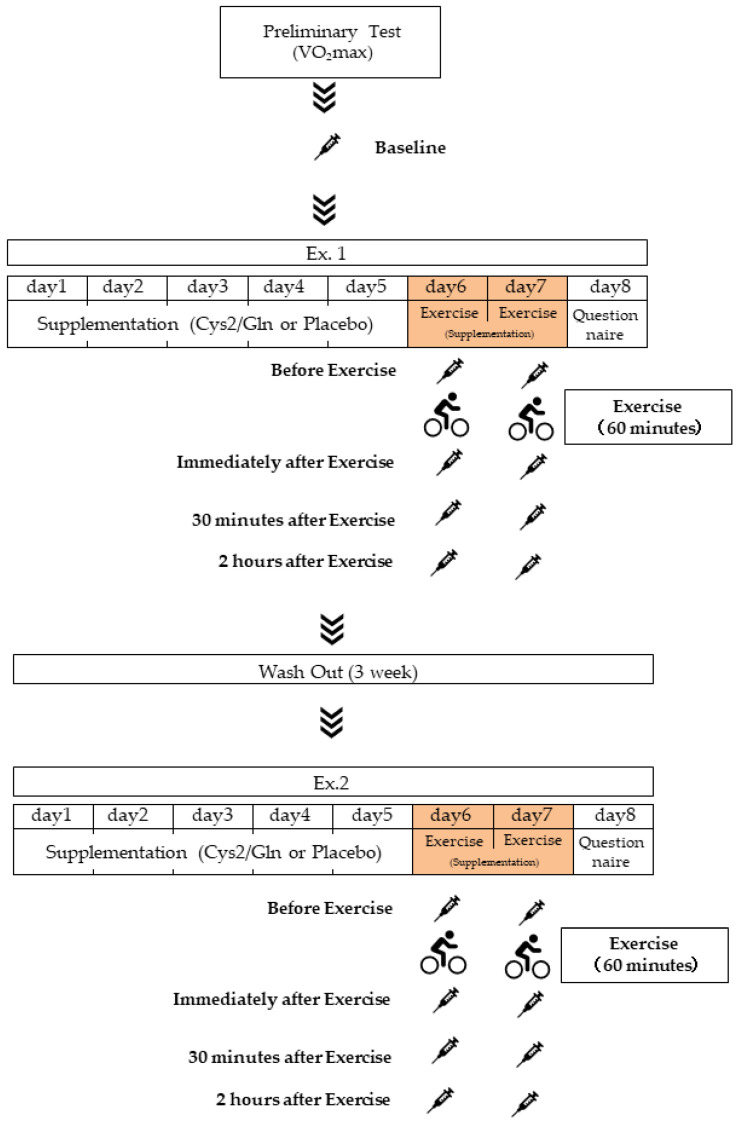
Experimental design. A preliminary test of baseline blood biochemistry and VO_2_max is carried out at first. Ex.1, Experiment 1. Ex. 2, Experiment 2. Cys2/Gln, cystine/glutamine mixture. A total of 16 subjects were recruited. For Ex. 1, 8 subjects were assigned randomly to the amino acid mixture trial (AAM Trial), the other 8 subjects were assigned to the placebo trial (PLA Trial), while for Ex. 2, subjects were re-assigned to the other trial.

We designed a double-blind, randomized, placebo-controlled crossover study. As shown in Figure 1, the participants were recruited to participate in two separate experimental trials with a 3-week wash out period: supplementation of either (1) Cystine/Glutamine mixture (Cys2/Gln) administration, the amino acid mixture trial (AAM trial) or (2) placebo protein trial (PLA trial). The whole experiment was carried out in a separated manner, characterized as Experiment 1 (Ex.1) and Experiment 2 (Ex.2). Protocols and flows of Ex.1 and Ex.2 were identical. After 5-day pre-exercise consumption, on the 6th and 7th day of each trial, participants were instructed to fast after 9:00 PM one night before and avoid water ingestion after 0:00 AM. Participants were then asked to urinate in the morning 2 h before arriving at the laboratory between 9:00 and 9:30 AM without consuming breakfast, urine and pre-exercise blood samples were then collected. Participants were then asked to consume the amino acid mixture or placebo with 200 mL water, rest in their seats for 30 min, and complete the questionnaire during the rest time. After a warm-up for 3 min at 60–90 W, the participants exercised on a cycle ergometer for 60 min at a target intensity of 70% targeted heart rate. Blood and urine were collected at the indicated time points (Figure 1). The experimental protocols were approved by the Ethics Committee of Waseda University (2016-288) and the Ethics Committee of Ajinomoto Co. (2016-33). Written informed consent was obtained from all participants prior to their enrollment in the study. The experiments were carried out from September 2017–February 2018. This study was registered in advance using the University Hospital Medical Information Network Center, a system for registering clinical trials (ID: UMIN000026008). During the experiment, ratings of perceived exertion (RPE) were obtained using the Borg scale [25]. A questionnaire for evaluation of the physical condition (QPC) was finished on the final day of each trial. Fatigue/muscle soreness, gastrointestinal symptoms, cold symptoms, and insomnia symptoms were answered using a 7-point scale, where 1 point means “absolutely no” and 7 points means “absolutely yes”.

### 2.2. Participants and Amino Acid Mixture Supplementation

In total, 16 young men were recruited to the study, 13 of them finished this study. The participants were recreationally active and had no chronic diseases. Participants read and signed an informed consent form prior to engaging in the study. Inclusion criteria were as follows: (a) participants had to be male students from Waseda University between the ages of 20 and 40 years old, (b) participants had to be healthy and free of any known disease determined by a medical history questionnaire, (c) not allergic to soy protein, (d) participants had to avoid the performance intensive training during the experimental period, and (e) participants had to abstain from non-steroidal anti-inflammatory drugs, gastrointestinal medicine, amino acid supplements, and alcohol for 3 days prior to participation. The amino acid mixture (2.46 g/pack, glutamine 1 g, cystine 0.23 g, and maltodextrin 1.23g in each pack) or the placebo (2.46 g/pack, maltodextrin 2.46 g in each pack) was consumed by the subjects 3 times per day (10:00 AM, 3:00 PM, and immediately before bedtime) for a consecutive 7 days during the experiment (Figure 1). On day 6 (1st exercise day) and day 7 (2nd exercise day), a consecutive two-day exercise experiment was carried out. The doses and regimens of glutamine and cystine were chosen based on previous studies that successfully observed a suppressive effect on oxidative stress without adverse effects in healthy adults [26,27,28].

### 2.3. Blood Sampling and Biochemistry Analyses

Venous blood samples were collected by venipuncture at the indicated time points (Figure 1). Blood samples were collected into ethylenediaminetetraacetic acid (EDTA)-containing tubes. The EDTA-containing tubes for plasma separation were immediately centrifuged at 3000 rpm (approximately 1700× *g*) for 10 min at 4 °C. Plasma samples were stored at −80 °C until analysis. Plasma total protein, albumin, albumin/globin ratio (A/G), total bilirubin, aspartate aminotransferase (AST), alanine aminotransferase (ALT), lactate dehydrogenase (LDH), γ-glutamyl transpeptidase (γ-GTP), creatine kinase (CK), amylase, total cholesterol (T-CHO), low-density lipoprotein cholesterol (LDL-CHO), high-density lipoprotein cholesterol (HDL-CHO), aldolase, triglyceride, free fatty acid, uric acid, blood urea nitrogen, creatinine, glucose, bile acid, blood concentration of calcium ion, chloride ion, potassium ion, sodium ion, and inorganic phosphorus were measured by Koutou-Biken Co. (Tsukuba, Japan). Glycerol, lactate, and ketone body concentrations were measured with an EnzyChrom™ Glycerol Assay Kit, EnzyChrom™ Lactate Assay Kit, and EnzyChrom™ Ketone Body Assay Kit (Bioassay systems, Hayward, CA, USA).

### 2.4. Reagents for the Cell Culture and Cell Experiment

Dulbecco’s modified Eagle medium (DMEM), Dulbecco’s phosphate buffered saline (DPBS) and penicillin–streptomycin–glutamine (100×) were obtained from Thermo Fisher (Waltham, MA, USA). Fetal bovine serum (FBS) was obtained from ICN Biomedicals (Costa Mesa, CA, USA). HEPES buffered saline solution was purchased from Lonza Ltd. (Walkersville, MD, USA). Trypsin-EDTA solution and L-cystine dihydrochloride were purchased from Sigma Chemical Company (St. Louis, MO, USA). Amino-acid-free DMEM was purchased from Nacalai Tesque (Kyoto, Japan). Sodium hydrogen carbonate (NaHCO_3_), hydrogen peroxide (H_2_O_2_), 100 *w*/*v*% trichloroacetic acid solution (TCA), and 1 mol/L hydrochloric acid (HCl) were purchased from Wako Pure Chemical Industries (Osaka, Japan). Reduced glutathione (GSH) and dichloromethane were obtained from FUJIFILM Wako Pure Chemical Corporation (Osaka, Japan).

### 2.5. Cell Culture and Oxidative Stress with H_2_O_2_ Stimulation

C2C12 myoblasts were purchased from KAC Co., Ltd. (Kyoto, Japan) and were grown at 37 °C and 5% CO_2_ in DMEM with 10% FBS, 1% penicillin–streptomycin–glutamine, and 1% HEPES. Myoblasts were seeded in XFp cell culture mini plates (Seahorse Bioscience, North Billerica, MA, USA) at 1.8 × 10^4^ cells/mL/dish. C2C12 myoblasts at approximately 80–90% confluence were differentiated into myotubes upon further incubation in 2% horse serum for 2–3 days. Differentiated C2C12 cells were incubated with 1/5 DMEM (DMEM medium diluted 5-fold with amino-acid-free DMEM or 1/5 DMEM supplemented with 1 mM Cys2-HCl) for 120 min. The cells were exposed to 0.12 mM of H_2_O_2_ with/without 1 mM Cys2-HCl for 60 min.

### 2.6. Fatty Acid Oxidation Assay and Mitochondrial Oxygen Consumption Rate

After exposure of H_2_O_2_ with/without Cys2 treatment, cells were washed twice with 1× KHB buffer (110 mM NaCl, 4.7 mM KCl, 2 mM MgSO_4_, and 1.2 mM Na_2_HPO_4_, pH 7.4, 2.5 mM glucose, and 0.5 mM carnitine) and incubated in a CO_2_-free incubator at 37 °C for 60 min. Following incubation, baseline measurements of the oxygen consumption rate (OCR) were recorded as an indicator of basal oxidative metabolism, using the Extracellular Flux Analyzers XFp (Agilent Technologies, Santa Clara, CA, USA). The palmitate–BSA conjugate was injected to a final concentration of 120 µM palmitate to measure the extracellular oxygen consumption rate for 80 min. The mitochondrial stress test was then performed to assess the bioenergetic status of the cells [26]. Cells were treated with oligomycin (a complex V inhibitor, final concentration 3 μM) at 20 min to induce maximal glycolytic metabolism, FCCP (carbonyl cyanide-4-(trifluoromethoxy) phenylhydrazone; uncoupling agent, final concentration 3 μM) at 50 min to uncouple electron transport and induce peak OCR, and antimycin A and rotenone (complex III and I inhibitors, respectively, final concentration 0.5 μM each) at 80 min to reveal non-mitochondrial respiration, after injection of the palmitate–BSA conjugate [29]. The maximal respiration after the addition of FCCP then reflects the maximal capacity of the electron transport chain [30]. The maximal respiration rate was calculated as the maximal OCR minus non-mitochondrial basal OCR determined after antimycin A and rotenone.

### 2.7. Sample Size Calculations and Statistical Analyses

The sample size was determined to be 16 based on our pilot study and a previous study [9]. The validity of this sample size was examined by calculating the statistical power. The sample size was validated using RPE as the primary study outcome, using the same sample size calculation methods used in a previous crossover study [9]. Analyses were performed using G*Power 3.1.0 (Heinrich-Heine-Universität Düsseldorf, Düsseldorf, Germany) [31]. Due to withdrawals, the final sample size was 13. We validated the achieved power based on our data. The effect size was set to 0.8 based on a calculation using means. For two trials with an alpha level set at 0.05 and a correlation of 0.5, a total sample size of 13 would provide 91% power to detect between-trial differences. All values are expressed as mean ± standard deviation. For human trials, a linear mixed model for repeated measures (intervention, time (treated as categorical data), interaction (between intervention and time), and period related to a crossover study design as fixed factors and participants as the random factor) was applied. VO_2_ during each trial was compared using a one-way repeated measures analysis of variance (ANOVA). A two-way repeated measures ANOVA was applied to determine the main effect of time and trial, and the interaction effect of time × trial on other variables. A linear mixed effect model was applied for the 2-trial comparison. For cell experiments, for comparison between the two groups, Sidak’s multiple comparison test following an analysis of variance test was used. For comparison among three groups, significant differences were determined using Tukey’s multiple comparisons test. Data were analyzed using SPSS Statistics, version 25 (SPSS Inc., Chicago, IL, USA) or GraphPad Prism 7.04 software (GraphPad Software Inc., San Diego, CA, USA), with *p* < 0.05 being considered significant. Part of data in free fatty acid analyses were treated as missing values by the analyzing software. For questionnaire analyses, there was no consideration for the multiplicity between evaluation outcomes, and between time points. All analyses in this study were planned and conducted after the unblinding for exploratory purposes.

## 3. Results

### 3.1. Physiological Variables throughout the Experiment

The characteristics of the participants were as follows: age, 22.2 ± 2.6 (mean ± standard error) years; height, 172.0 ± 4.6 cm; body mass, 63.5 ± 8.8 kg; heart rate max 186.9 ± 9.3 bpm; and maximal oxygen uptake (VO_2_ max), 42.8 ± 9.6 mL·kg^−1^·min^−1^.

### 3.2. A 7-Day Amino Acid Supplementation Decreased Ratings of Perceived Exertion

**Figure 2:**
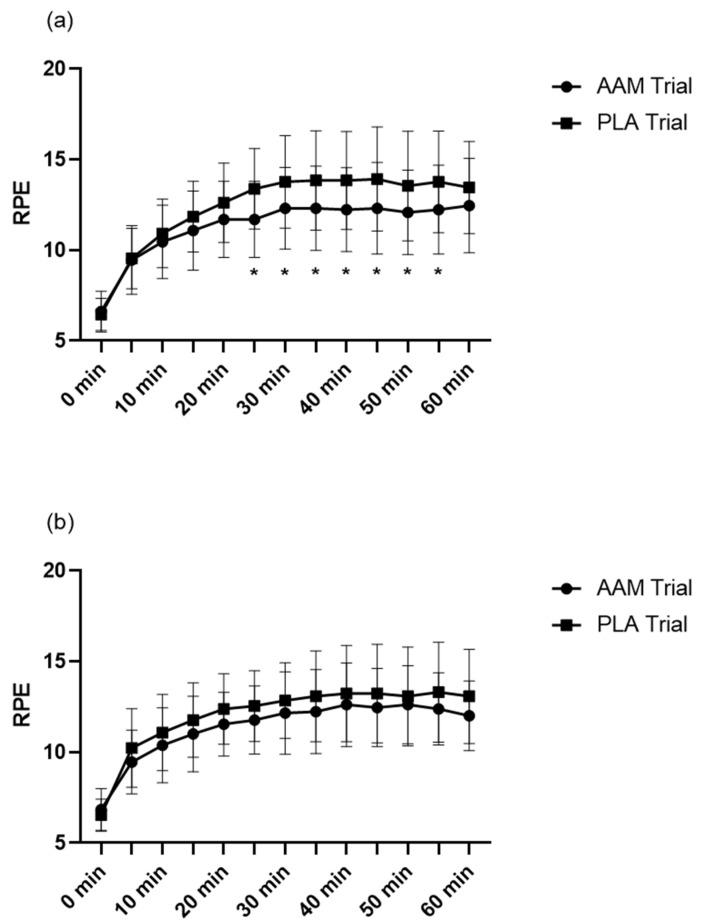
Ratings of perceived exertion throughout the experiment. (**a**) Results of RPE on the 1st exercise day (day 6) and (**b**) results of the 2nd exercise day (day 7). RPE, ratings of perceived exertion. AAM trial, amino acid mixture trial. PLA trial, placebo trial. Pre, before exercise. Post, immediately after exercise. Post 30 min, 30 min after the exercise ends. Post 60 min, 60 min after the exercise ends. * *p* < 0.05. A linear mixed model for repeated measures (intervention, time (treated as categorical data), interaction (between intervention and time) and period related to a crossover study design as fixed factors and participants as the random factor) was applied for the two intervention comparison (total *n* = 13).

We employed the Borg Scale to evaluate RPE, an index of fatigue by self-reporting. RPE was measured every 5 min during each 60 min exercise test. As shown in Figure 2, on both days 6 and 7, after the exercise test started, participants in the AAM trial reported a lower RPE index at the timepoints of the 25th, 30th, 35th, 40th, 45th, 50th, and 55th minutes (*p* < 0.05).

### 3.3. A 7-Day Amino Acid Supplementation Enhanced Fatty Acid Utilization

**Figure 3:**
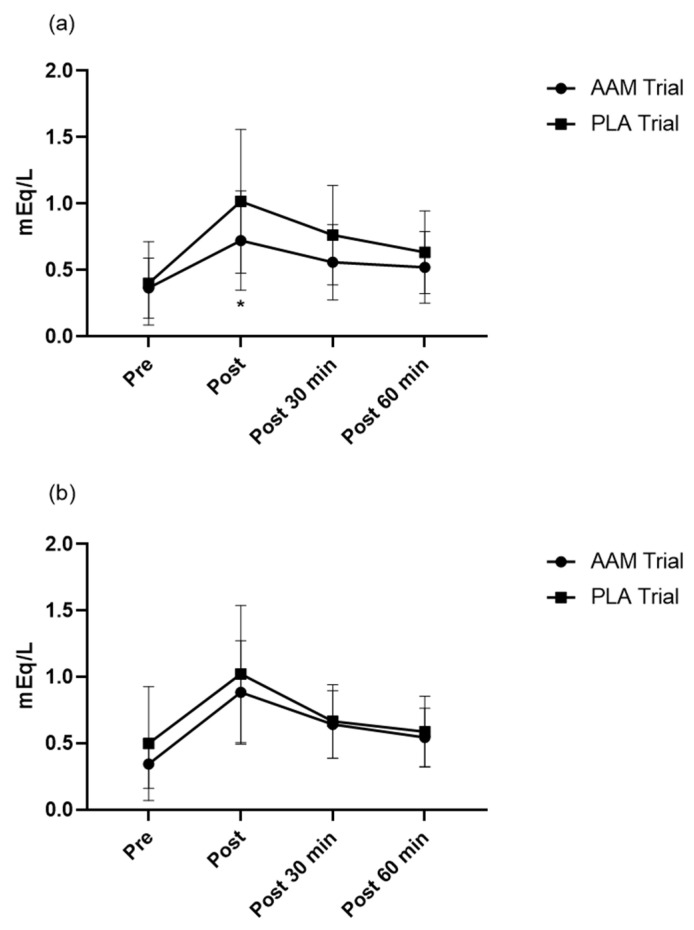
Profiles of plasma free fatty acids throughout the experiment. (**a**) Results of 1st exercise day (day 6) and (**b**) results of the 2nd exercise day (day 7). FFA, free fatty acid. AAM trial, amino acid mixture trial. PLA trial, placebo trial. Pre, before exercise. Post, immediately after exercise. Post 30 min, 30 min after exercise ends. Post 60 min, 60 min after exercise ends. * *p* < 0.05. A linear mixed model for repeated measures (intervention, time (treated as categorical data), interaction (between intervention and time), and period related to a crossover study design as fixed factors and participants as the random factor) was applied for the two intervention comparison (total *n* = 13).

We measured baseline and post-exercise plasma free fatty acid concentrations at several timepoints in both trials. As shown in Figure 3, on the 1st exercise day (day 6), immediately after exercise, plasma free fatty acid is significantly lower in the AAM trial than that in the PLA trial (*p* < 0.05). We also measured glycerol and ketone bodies, which are metabolites of triglyceride, but no significance was observed between trials (data was submitted as Supplementary Table S1).

### 3.4. Effects of Exercise and Amino Acid Supplementation on Blood Biochemistry and the Subjective Physical Condition

We measured various blood biochemistry markers in all trials. Plasma lactate, total protein, albumin, A/G, total bilirubin, AST, ALT, LDH, γ-GTP, CK, amylase, T-CHO, LDL-CHO, HDL-CHO, aldolase, triglyceride, uric acid, blood urea nitrogen, creatinine, glucose, bile acid, blood concentration of calcium ion, chloride ion, potassium ion, sodium ion, and inorganic phosphorus were not altered by exercise or amino acid supplementation (data are submitted as Supplementary Table S1).

### 3.5. Effect of Cys2 on Mitochondrial Respiration and Fatty Acid Utilization in C2C12 Myotubes

**Figure 4:**
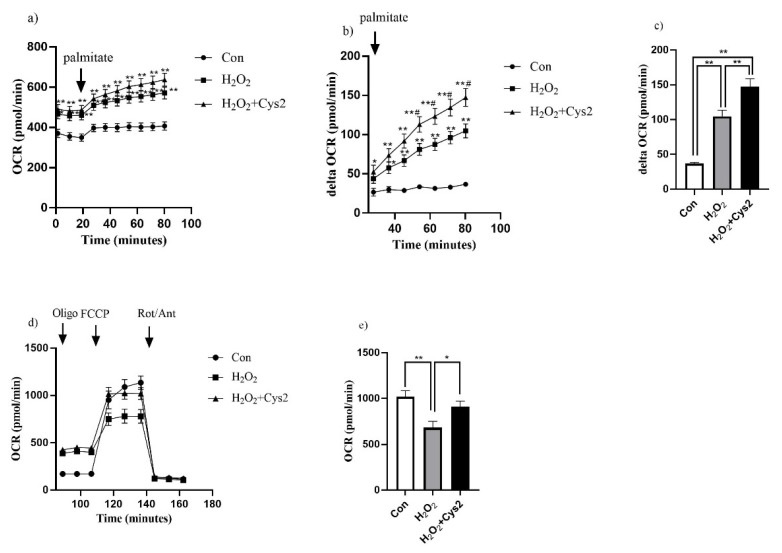
Effect of Cys2 on mitochondrial oxygen consumption and fatty acid utilization after H_2_O_2_ treatment. (**a**) Oxygen consumption ratio (OCR), (**b**) time-course changes in delta OCR after injection of the palmitate–BSA conjugate, (**c**) delta OCR at 80 min after injection of palmitate–BSA conjugate, and (**d**,**e**) time-course changes in OCR and mitochondrial maximal respiration after injection of the palmitate–BSA conjugate. Data are shown as the mean ± standard error (*n* = 10–18/group). * *p* < 0.05; ** *p* < 0.01 (vs. control), # *p* < 0.05 (vs. H_2_O_2_). Con, incubation in 1/5 DMEM without H_2_O_2_ exposure; Oligo, oligomycin (a complex V inhibitor); FCCP, carbonyl cyanide-4-(trifluoromethoxy) phenylhydrazone; uncoupling agent); Rot/Ant, antimycin A and rotenone (complex III and I inhibitors, respectively).

Following the injection of the palmitate–BSA conjugate, OCR significantly increased in the H_2_O_2_ and H_2_O_2_ + Cys2 groups compared with that of the control group (Figure 4a). Additionally, delta OCR significantly increased in the H_2_O_2_ and H_2_O_2_ + Cys2 groups compared with that in the control group (*p* < 0.01) (Figure 4b,c). Furthermore, the H_2_O_2_ + Cys2 group was significantly increased compared with that of the H_2_O_2_ group (*p* < 0.01, Figure 4b,c). Moreover, we measured the mitochondrial maximal respiration in the medium added with the palmitate–BSA conjugate. The H_2_O_2_ group showed significantly decreased mitochondrial maximal respiration compared with that of the control group (*p* < 0.01), and the H_2_O_2_ + Cys2 group showed a significant increase in mitochondrial maximal respiration compared with the H_2_O_2_ group (*p* < 0.05. Figure 4d,e).

## 4. Discussion

Exercise triggers various physiological responses in the body. Though regular exercise training favors the redox system, thus helping to maintain cellular homeostasis, such as by increasing the levels of anti-oxidative molecules, such as Sirtuin 1 (Sirt1), and endogenous histone deacetylase inhibitor, such as β-hydroxybutyrate, acute bouts of strenuous exercise may induce unfavored oxidative stress [32,33]. Therefore, anti-oxidative supplementations are being used extensively between athletes and sport-active populations.

Glutamine has been used as a sport supplementation for decades due to its outstanding anti-oxidation properties. Acute or chronic glutamine supplementations have been widely applied to take advantage of its antioxidant, anti-inflammatory, and immuno-regulating capacity. Recently, Caris and colleagues reported that 6-day glutamine (20 g/day) supplementation enhances in-trial glycemia and attenuates the increase in RPE (*n* = 9, treadmill exercise to exhaustion, 70% VO_2max_) [34]. Cystine is an oxidized form of cysteine. In 2018, a review summarized the potential health-promoting effects of cystine, that are supported by clinical evidence, including protective effects toward digestive diseases, the alleviating role in chronic inflammation by acting as a crucial antioxidant, controlling glycaemia, and vascular inflammation [35]. Fatty acids are important respiratory substrates during intensive cellular respiration activities, for instance, under the context of physical activity. Meanwhile, beta-oxidation, which is the main form of FAO, is in the mitochondrial matrix [36]. Enhancing the total capacity of FAO by increasing mitochondrial respiration had proved to be an applicable way to enhance exercise capacity [37]. In the present study, we discovered that Cys2/Gln supplementation showed an increasing effect on FAO, in human trials. To investigate whether and why cystine can enhance fatty acid utilization rate, we built a H_2_O_2_-activated C2C12 myotube model, which is employed as a model of an exercise-induced oxidative environment, the supplementation of cystine significantly enhanced mitochondria respiration and fatty acid uptake. To our knowledge, this is the first study demonstrated that cysteine/cystine is reported for its effects on enhancing fatty acid utilization energy production, therefore, the mechanism of cysteine/cystine’s health-promoting capacities is unveiled further.

In other studies, it has been shown that cysteine supplementation can significantly reduce the activation of nuclear factor-kappa B (NF-κB) in activated human THP-1 (macrophage) cell lines [38], as well as significantly inhibit the expression of an intercellular adhesion molecule (ICAM)-1 [39] and production of IL-8 in both the THP-1 cell line and peripheral blood mononuclear cells (PBMCs) [38]. The authors also mentioned the possibility of amplifying the effects of cysteine by combining it with other nutrients, such as theanine, to achieve a maximal effect [40,41,42,43,44,45]. Previous research also pointed out that 10day supplementation of 700 mg cysteine combined with 280 mg of theanine supplementation ameliorated the inflammation response after a prolonged period of intense exercise (*n* = 15, an average total distance of 152.2 km), characterized by unaffected high-sensitivity C-reactive protein (hs-CRP) level, neutrophil count, and lymphocyte count, compared to the placebo takers [46]. However, we did not observe a significant anti-inflammatory or anti-oxidation effect of Cys2/Gln supplementation in our experimental model (data accessible as Supplementary Table S1), whether Cys2/Gln supplementation can exert positive effects in humans during exercise should be further studied using various experimental models to obtain convincing results.

Cysteine is also a building material of glutathione, while glutathione plays key roles in antioxidant defense, nutrition metabolism, regulation of cellular events, including gene expression, signal transduction, cytokine secretion, and immune response [47]. Homeostasis of glutathione has great implications for health, as well as athletic condition [45]. Endurance-exercise-induced lipid peroxidation can be minimized by glutathione supplementation [42], while glutathione supplementation is reported to suppress muscle fatigue induced by prolonged exercise in both animal and human models [48]. In the indicated study, the peroxisome-proliferator-activated receptor (PPAR)-γ coactivator-1α protein and mitochondrial DNA levels were significantly higher after glutathione supplementation (25% and 53% higher, respectively) in a murine model [48]. Meanwhile, the elevation of blood lactate was suppressed by glutathione supplementation (15% decreased compared to the placebo group). It is supposed that cystine supplementation may alter the over-consumption of glutathione during endurance exercise by increasing bioavailable cysteine, contributing to biosynthesis of glutathione, ensuring the capacity to quench free radicals, therefore, ameliorating fatigue and enhancing exercise performance and athletic well-being [46]. In addition, it is demonstrated that oxidative stress could alter membrane permeability and influence Ca^2+^ homeostasis, therefore inducing mitochondrial DNA mutations, and damaging the mitochondrial respiratory chain [49], while the antioxidative properties of cysteine and glutamine, as well as their consequent GSH, are potential rescuers upon mitochondrial dysfunction triggered by oxidative stress during exercise.

We admit that the present study has limitations. Firstly, on the 1st exercise day (day 6), RPE is significantly reduced and FFA oxidation is significantly improved; while on the 2nd exercise day (day 7), the 2nd day of the consecutive 2 days of exercise, though an ameliorating trend is still observed, no significance is found, which is in accordance with the FFA profile, where no significant difference is observed. The anti-fatigue properties created by a 7-day supplementation may be not enough for repeated exercise, a longer period of taking the supplementation may receive more powerful effects toward exercises performed on consecutive days. A recent study also argues that the state of energy and fatigue might be independent, therefore, introduction of the measurement on both energy and fatigue using unipolar measurement tools may provide new insights [50]. Secondly, we did not manage to observe anti-inflammatory effects of Cys2/Gln supplementation, despite the anti-inflammatory properties of the above amino acids being reported extensively. Due to our limited sample size and non-completion by some subjects, an enlarged sample size may be satisfying.

In brief, in the present study, we adopted a 60 min exercise test to validate the multi-effects of a 7-day Cys2/Gln supplementation during and post-endurance exercise. The combination of cysteine/glutamine exerts a more powerful capacity on mobilization and oxidation of free fatty acids, therefore, alleviating fatigue. Further studies are encouraged to employ maximal or submaximal exercise capacity tests to examine the potential roles and possible mechanisms of Cys2/Gln supplementation on endurance.

## 5. Conclusions

A 7-day Cys2/Gln supplementation showed alleviating effects on fatigue by reducing the self-reporting fatigue index in a randomized, placebo-controlled, crossover trial. By using a C2C12 cell model, we showed that the mechanism may be associated with increased fatty acid utilization by enhancing mitochondrial respiration in the muscle.

## Supplementary Materials

The following are available online at https://www.mdpi.com/article/10.3390/sports10100147/s1, Table S1: Effects of Exercise and Amino Acid Supplementation on Blood Biochemistry Parameters.
